# Living with COPD: understanding patient experiences through the lens of photovoice

**DOI:** 10.1186/s12890-023-02738-4

**Published:** 2023-11-09

**Authors:** Jennifer Sumner, Anjali Bundele, Ravi Shankar, Lin Siew Chong, Yanika Kowitlawakul, Amartya Mukhopadhyay

**Affiliations:** 1 Medical Affairs – Research, Innovation & Enterprise, Alexandra Hospital, National University Health System, Singapore, Singapore; 2 Yong Loo Lin School of Medicine, National University of Singapore, Singapore, Singapore; 3 Chief Operating Office, National University of Singapore, Singapore, Singapore; 4 School of Nursing, College of Public Health, George Mason University, Fairfax, VA USA; 5 Division of Respiratory and Critical Care Medicine, Department of Medicine, National University Hospital, Singapore, Singapore

**Keywords:** Qualitative research, Photovoice, Chronic obstructive pulmonary disease, Lived experience

## Abstract

**Background:**

Conventional quantitative or qualitative methodologies may not encompass the wide array of experiences of individuals living with Chronic Obstructive Pulmonary Disease (COPD). We used a novel approach – photovoice—to understand the impact of COPD on activities of daily living (ADLs) in a multicultural Asian country.

**Methods:**

We recruited a purposive sample of eight patients from the outpatient clinics of the National University Health System, Singapore, between December 2020 and August 2021. We adopted a photovoice approach for data collection; participants were invited to take photos of how ADLs were impacted by COPD and attend a follow-up interview. An Interpretative Phenomenological Analysis method was used to analyze the data. Data saturation was reached by the seventh patient.

**Results:**

COPD and the resulting breathlessness had a profound and diverse impact on our participants’ lives. Living with COPD required substantial changes to how everyday tasks are performed, and participants learnt new strategies to deal with such tasks. A mixture of active and passive coping styles was evident. Feelings of frustration, anxiety and a sense of isolation were also reported. Contextual factors impacting ADLs included challenging climatic conditions and the local popularity of traditional or alternative medicine.

**Conclusion:**

The photovoice technique improved our understanding of the lived experiences of COPD patients and can benefit those who struggle to articulate their views by offering a different way to communicate beyond conventional interviewing.

**Supplementary Information:**

The online version contains supplementary material available at 10.1186/s12890-023-02738-4.

## Introduction

Patients with Chronic Obstructive Pulmonary Disease (COPD) suffer from a range of debilitating symptoms, including breathlessness, cough and sputum production, poor sleep, depression, and skeletal muscle loss—such symptom burden can pose a significant challenge to activities of daily living (ADLs) [1–5]. Patient-centric symptom control is the cornerstone of COPD management. However, disease management is complex because of the variability of symptoms, poor correlation between the severity of COPD and lung function tests, and the progressive nature of the disease. For instance, the time of day, type of activities undertaken, and weather conditions can all impact symptom severity, making it highly unpredictable and difficult to manage [4, 6]. As a result, patients often plan their daily activities in advance, contributing to a significant disease burden [7] and reducing quality of life [4, 8, 9].

Qualitative interview studies have explored the impact of COPD on the lived experiences of patients [5, 6, 10–12]. However, these traditional interview approaches have limitations. First, the meaning and significance of the participants’ experiences may not be fully captured in interviews if reflexivity in the research is not properly considered [13]. Furthermore, the power imbalance between interviewer and interviewee may leave participants feeling intimidated and unwilling to share, reducing the authenticity and accuracy of the data gathered [13]. Lastly, traditional interview approaches can be challenging for those who struggle to articulate complex issues or have physical limitations which limit verbal communication [14, 15]. A different approach – *Photovoice*—may offer a solution for capturing the patient’s perspective.

Photovoice uses a blend of photographs (taken by the participant) and words to explore issues, perspectives, and experiences with participants [16]. Participants are empowered through photo taking by deciding what images they wish to capture and discuss. It also allows participants to express themselves in a form that may otherwise be hard to explain with words alone [17, 18]. Traditionally, photovoice has been used to encourage those of different social classes, ages or cultural backgrounds to raise overlooked issues, which promoted dialogue and solved community problems [16, 19–21]. Today, photovoice is used in a broad array of topics.

While much work has gone into using participatory approaches to explore the lived experiences of those with COPD [4, 5], photovoice has rarely been used [22]. We posit that this novel approach to data collection will have several benefits. First, the methodology can serve as a form of validation by reinforcing the reliability of the established findings and reducing method-specific bias. The methodology also provides an innovative approach to gathering and analysing data, which may result in a deeper comprehension of the subject, enhanced data collection efficiency, and increased accessibility for participants [14, 15, 23, 24]. Therefore, the objective of this study was to use a different methodological approach (photovoice) to understand the impact of COPD on ADLs.

## Methods

We recruited a purposive sample of COPD patients attending the outpatient clinics of the National University Health System, Singapore, between 1^st^ December 2020 to 24^th^ August 2021. We deliberately included individuals from diverse ethnic backgrounds, varying grades and durations of COPD, and different treatment modalities (including those on oxygen) to enhance the representativeness of our sample. Inclusion criteria included adult participants (≥ 21 years), who owned a camera phone and had a clinical diagnosis of COPD (any grade). Patients with a diagnosis of dementia, poor cognition, or medical conditions impacting mobility (e.g., recent surgery) were excluded.

The treating physician identified potential participants during routine outpatient visits and subsequently referred them to the research team. A research assistant then introduced the project to the participants, and if they expressed interest, informed consent was obtained.

### Photovoice approach

#### Step 1: Baseline interview

Following consent, participants attended a baseline interview with a research assistant [LSC, AB]. The purpose was to build rapport with the participant and educate them on photo taking. During the interview, the project’s aims were explained with a pictorial reference card. The card included an overview of what was meant by activities of daily living, instructions on photo taking and how to send the pictures or messages to the research team. Demographical data were also collected. If requested, participants’ carers also attended the training session and supported participants during the study.

#### Step 2: Photo taking

Following Step 1, participants were given two weeks to take at least five photographs. Participants were informed that while there was no set limit on the number of photos they could capture, it might not be feasible to discuss each one in detail during the follow-up interview. Participants were advised to send photos directly to a dedicated mobile number managed by the research team.

#### Step 3: Follow-up interview

After two weeks, participants were invited back for a single interview to discuss their most important pictures. The interviews were conducted by two female health services researchers, trained in qualitative interview techniques [LSC (MSc) and AB (MPH)]. The interview guide followed the SHOWeD approach [16]. For each photo, the participants were asked: What do you See here? What is really Happening here? How does this relate to Our lives? Why does this problem exist? What can we Do about it? The interviews were conducted in English or Chinese by a native speaker according to the participant’s preference. Following the interviews, interviewers wrote memos to capture their immediate reflections, insights and take aways. Interviews lasted for approximately 45 min. All interviews were audio recorded and later transcribed. Chinese transcripts were translated to English by a native speaker and verified by a second native speaker for accuracy. Discrepancies were discussed and resolved by mutual consensus.

### Analysis

Interviews were analysed using an interpretive phenomenological analysis (IPA) approach [25], which covers an individual's opinion regarding specific events or experiences and intends not to produce an objective truth or theory. Since the complete interpretation of the participant’s world is impossible, researchers must also use their judgement [25]. Hence the analysis is interpretative in addition to being phenomenological. The analysis followed a bottom-up approach to generate themes from the interviews. Three coders used Microsoft Word to develop an initial coding structure on one transcript as a group (JS, AB, LSC). Coding for the remaining transcripts was then completed individually, followed by group discussion and alignment on coding decisions. Following this, codes were grouped into sub-themes and themes, through group discussion (JS, AB, LSC, RS) [26]. Data saturation was reached by the seventh patient.

### Reflexivity

We recognise that researchers’ backgrounds, biases, and experiences could influence the study [27]. To address this, we adopted several approaches throughout the study. Firstly, interviews followed the SHOWeD approach; this framework enables participants to lead the discussion, minimising interviewer bias. In addition, memo writing after each interview allowed interviewers to reflect on their thoughts and consider biases. Finally, regular group discussion during the coding process helped to collectively maintain a more neutral and empathetic stance in understanding the participants’ experiences. Our reflexive approach helped ensure the integrity and rigour of our project.

## Results

The study adheres to the COREQ (Consolidated criteria for reporting qualitative research) checklist for reporting [28]. We approached fifteen participants to participate in the study, of which eight completed the study and seven did not (two were uncontactable, four were no longer interested, and one passed away before the study ended). Participants were all male, the majority had primary education or less, and had some problems with mobility, self-care, and their usual activities (Table 1).

**Table 1:** Participant characteristics

Characteristics	*N* = 8
Mean age: years (SD)	69.62 (6.54)
Sex: Male, n (%)	8 (100)
Ethnicity, n (%)	
Chinese	6 (76)
Malay	1 (12)
Indian	1 (12)
Marital status, n (%)	
Married	7 (88)
Divorced	1 (12)
Highest educational attainment, n (%)	
None or primary school level	4 (50)
Secondary school level	3 (38)
Diploma or higher	1 (12)
Employment status, n (%)	
Employed (full-time/part-time)	4 (50)
Retired	4 (50)
EQ5D, mean VAS score, mean (SD)	61.87 (15.56)
Any problem in EQ5D domains^a^, n (%)	
Mobility	6 (75)
Self-care	5 (63)
Usual activities	5 (63)
Pain and discomfort	4 (50)
Anxiety/Depression	2 (25)

*Abbreviations*: *SD* Standard Deviation, *VAS* Visual Analogue Scale

^a^Any problem is defined as scoring 2–5 on individual domains

The following four themes emerged from the data: i) breathlessness pervades everything, ii) benefits and barriers to lifestyle change, iii) social isolation and stigmatization, and iv) financial burdens. A summary of the findings is reported as follows and Table 2 includes supporting quotes from the interviews.

**Table 2:** Themes and sub-themes with supporting quotes

Theme and sub-theme	Example quotes from the interviews
Theme 1: Breathlessness pervades everything	*ID04: “Every morning when I have to go to work I have to wear socks… when I wear socks, I have to bend down, so every time I don't know why my chest my lungs, aahh…. once I'm done I'll wait for a while.”*
	Sub-theme 1: Challenging environment *ID01: “The weather on most days is hot and humid in Singapore…so it is a must to shower daily…however, it is challenging to shower for someone with COPD.”* *ID02: “I frequently look at the- air quality especially when its hazy outside…I also take a note of the time it is going to rain… I go out when air quality is within safe limits.”*
	Sub-theme 2: Learning from experience *ID07: “I’ve been like this for a few decades…so I know how to control my life.”* *ID05: “When it comes to eating, I now notice what I can and cannot eat, and which foods have or do not have an effect on me. I will take note of and remember if a type of food has an effect on me and not eat it again.”*
	Sub-theme 3: Developing treatment strategies *ID01: “Swallowing nest soup has beneficial effect on lung capacity…so I consume it very frequently…I also take supplements. I drink warm water every morning, put turmeric and honey and black seed oil…every morning I take this and I think it helps me.”*
Theme 2: Benefits and barriers to lifestyle change	*ID07: “I do some small light exercises in morning after getting up every day. It is very helpful for me. After exercising I gain more strength- there seems to be more air.”* *ID02: “Our domestic helper is meant to do household chores and not to provide care for me. So, I have to manage on my own.”* *ID07: “I decided to make some adjustments in my lifestyle… however, once in a while, there is still a temptation to pick up a cigarette or pour myself a glass of whiskey.”* *ID03: “I wanted to quit smoking initially…for a while I did quit, but I was still breathless. So, I felt that I am already in my seventies and …I do not have much else to do apart from smoking as my only hobby so I just chose to smoke.”* *ID01: “Sometimes people don’t want to help you…You call anyone, all working”*
Theme 3: Social isolation and stigmatization	*ID06: “When I walk with bigger steps…I’ll be breathless. I’m not breathless at home as no one is watching…I can walk slowly.”* *ID08: “I used to run every week. Now I can't even walk. That's the biggest problem. I can't exert myself, I can't perspire. I was in the army, so every week I used to run, now I can’t do that anymore…So I regret it. Because of my smoking.”* *ID04: “Most people don’t understand my health conditions and judge me…they do not understand my disability and think that I am lazy to lift heavy items”*
Theme 4: Financial burdens	*ID03: “Earlier, I got SMF [Seniors Mobility and Enabling Fund] assist for maybe 65% of the cost, now people like us do not get SMF anymore. I worry about exploiting my MediSave soon [National Healthcare insurance]. People like me, anytime when got infection must go hospital, the costs of even 2–3 days of medication is above a few thousand dollars.”* *ID07: “Actually my plan was to work till sixty-five and then retire…but because of this illness, I quit two years earlier. The biggest impact of quitting earlier is that, there’s no income.”*

### Theme 1: Breathlessness pervades everything

**Fig. 1:**
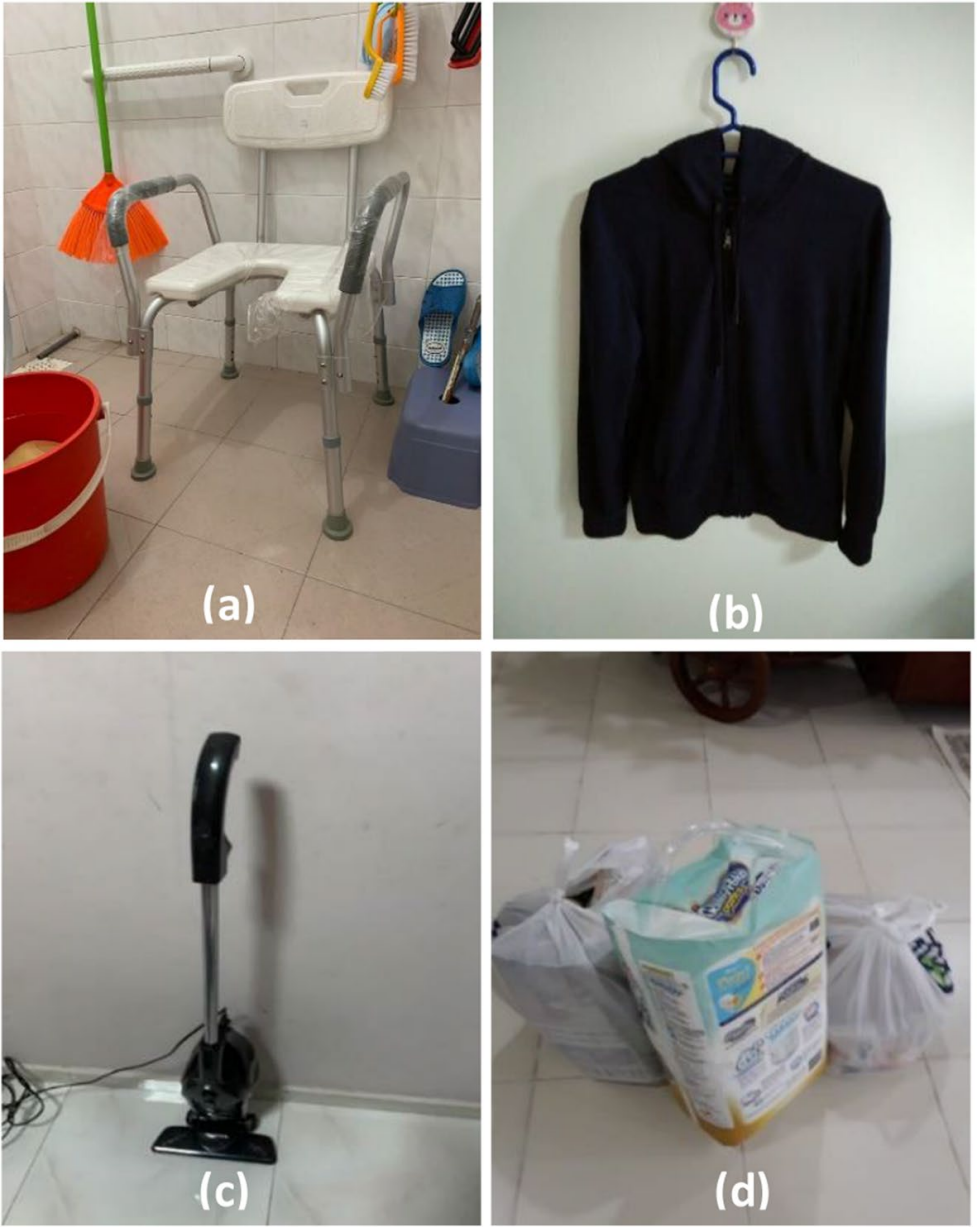
Examples of routine activities causing breathlessness, such as **a** showering, **b** dressing, **c** vacuuming, **d** and carrying heavy items from the shops

Most interviewees reported how breathlessness impacted all aspects of life, even routine daily tasks such as dressing, cooking and household maintenance (Fig. 1). Episodes of breathlessness ranged in severity, from transient and easily manageable to more severe incidences. Under the umbrella of breathlessness, three sub-themes emerged.

#### Sub-theme 1: Challenging environment

The local tropical environment frequently impacted our participants. References to the heat and humidity, air quality, and ventilation of indoor and outdoor spaces were made by most. Many avoided venturing outside on sunny days as they found it too challenging. Participants also expressed that walking outside was further compounded by the need to shower afterwards—another difficult task for them (Fig. 1).

Ventilation within indoor and outdoor spaces caused problems for many participants. Most public indoor places are air-conditioned; consequently, some were reluctant to visit them as it exacerbated their symptoms. This made tasks like purchasing everyday essentials difficult. The perspectives on naturally ventilated spaces were split. Some found it problematic, while others preferred the fresh outdoor air. Regardless of opinion, air quality was another factor participants were mindful of and most monitored air quality before going out. Poor air quality was particularly impactful on symptoms during the ‘haze season’ arising from regional forest fires [29].

**Fig. 2:**
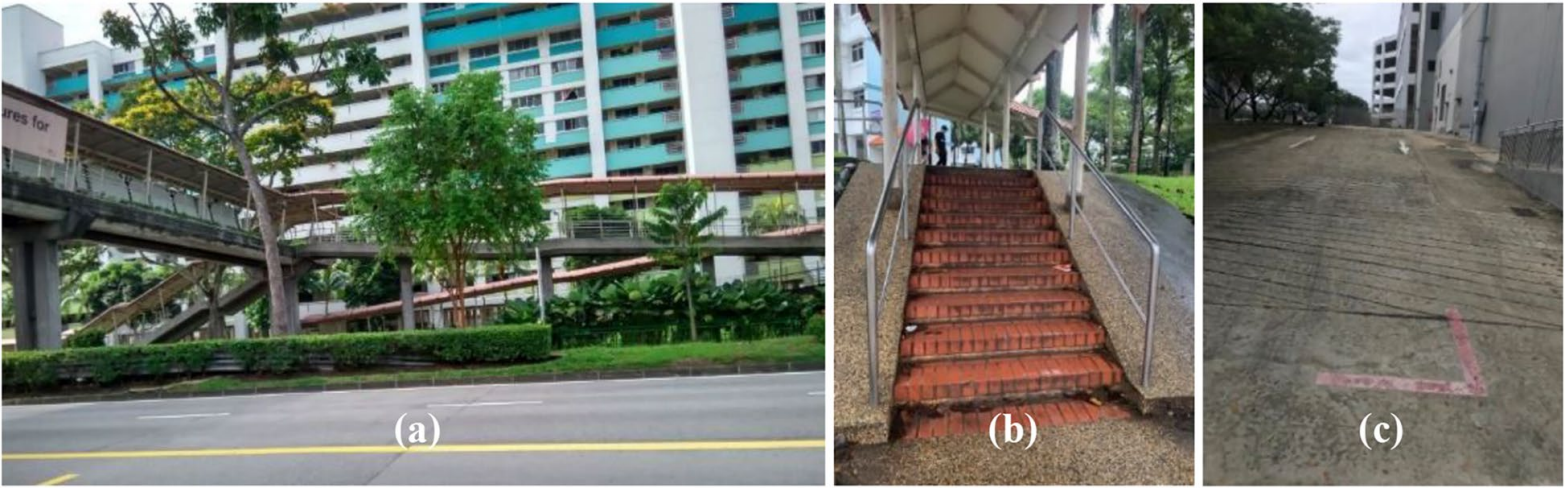
Images of infrastructural issues, including **a** the absence of lifts at overhead bridges, **b** staircases up to residential blocks, **c** slopes and undulating terrain

Other environmental challenges related to the physical infrastructure in Singapore (Fig. 2). Most participants found stair climbing hard and noted a lack of levelled walkways and crossings, which made walking difficult.

#### Sub-theme 2: Learning from experience

Most participants stressed that with experience, it is possible to adapt and get better control of their lives despite the limitations caused by COPD. Interviewees cited unique coping strategies, such as adopting lighter exercises, choosing appropriate foods to avoid exacerbation, breaking routine activities into smaller and manageable sub-activities, adjusting oxygen intake while performing certain activities and identifying the best body positions and postures – all to avoid being short of breath.

#### Sub-theme 3: Developing treatment strategies

In addition to adapting their everyday routines to avoid breathlessness, participants reported using alternative therapies and sources of advice to treat their condition. For example, the use of Traditional Chinese Medicine.

### Theme 2: Benefits and barriers to lifestyle change

Most interviewees perceived a positive relationship between a healthy lifestyle and improved ADLs, which motivated them to be healthy. Conversely, some reflected that there were times when they were tempted to revert to old habits or couldn’t see the benefit of changing their ways. Other challenges to a healthy lifestyle included a lack of support from others and technology barriers, which prevented them from accessing available resources.

### Theme 3: Social isolation and stigmatization

Participants described feelings of anger, helplessness and anxiety that affected many aspects of life. These feelings and their sense of control over their symptoms appeared to vary by location. For example, one participant reported how anxious they were travelling on the Mass Rapid Transit system (MRT), noting that they feared having an exacerbation while trapped underground. Another found the contained home environment made them feel more able to handle their physical limitations.

Some felt that they were judged as being responsible for their condition, especially when the participants smoked. As a result, they felt stigmatized and were cautious about seeking help, such as when lifting heavy shopping items, due to a fear of being belittled. Many also expressed that COPD had affected relationships with their spouse, friends, and family members leading to social isolation. Some highlighted that their lives had lost meaning as they could no longer enjoy activities, they took for granted before being diagnosed with COPD.

### Theme 4: Financial burdens

A lack of income support and social security was mentioned in some interviews. For instance, there was a perception, by some, that income support was only available for lower-income families, leaving others vulnerable to financial hardships. Financial worries also stemmed from their limited employment options, due to their physical disability. Some had to quit their jobs earlier than desired as they were not physically able. In contrast, others could not afford to retire early as they needed to financially support themselves and their families.

## Discussion

We found that the impact of COPD on the lives of our participants was diverse and complex, requiring substantial changes to facilitate everyday tasks and the development of coping strategies. Many of the lived experiences reported in our photovoice project were similar to those reported in conventional qualitative studies (e.g., interview-only approach) [4–6, 10–12]. The pervasive role of breathlessness, financial difficulties, and mental health issues are often described by patients and exemplify the multifaceted challenges COPD patients face. Through photovoice, we were able to amplify the voices of these patients and shed light on the interconnectedness of these themes, providing a holistic understanding of their experiences.

While many of the findings reflect existing literature, there were nuances in our data that are worth mentioning. Environmental-related symptom exacerbation is a well-known phenomenon in COPD [30, 31]. However, the combination of local climatic conditions particularly burdened our participants, including the tropical climate, which requires frequent showering [32], moving between air-conditioned and naturally ventilated environments, and poor air quality, exacerbated by hazy seasons [33]. It is important to recognize that with climate change, the challenges posed by the environment are likely to be exacerbated worldwide [34], and health systems must prepare for this.

Mental health was another significant barrier to ADLs. However, in our interviews, the topic emerged more indirectly, a well-known phenomenon in Asian cultures. For instance, individuals of Chinese descent may express their mental health issues more somatically [29]. East Asian cultures may also be less inclined to acknowledge symptoms due to the greater mental health stigma compared to Western counterparts [30]. Thus, culturally sensitive screening approaches must be adopted to account for differences in expression [29], and clinicians must use nuanced interviewing techniques during clinical follow-ups.

Faced with the many challenges of living with COPD, participants often reflected on their coping strategies. This ‘evolution of expertise’ has been reported in other chronic conditions [35] and reflects an active coping style or problem-solving approach. Active coping is associated with better health-related quality of life and mental health [36–39]. We also found instances where participants had poorly adjusted to their circumstances, exhibiting passive coping styles such as avoidance. Passive coping styles are problematic because they are associated with lower quality of life [36–39]. Referral to COPD rehabilitation is one way patients can be offered psychosocial support and training to build self-management capabilities [3]. Rehabilitation can enhance active coping styles [40], but the impact is limited by poor uptake of these programs [41].

The strength of this research lies in the photovoice approach. For participants with physical limitations or for those who find it hard to articulate, photovoice offers an alternative. Photovoice is also more empowering than traditional interviewing, as participants steer the conversation through their photograph selection. Despite the strengths of photovoice, there are limitations [16]. The photo taking activity may bias sampling to those able or willing to photograph. Photos may also not capture the full complexity of a situation and may have limited generalisability to other populations or contexts. Finally, photovoice can be a time-consuming and resource-intensive process, requiring access to cameras, training of participants, and adequate time for photo taking and analysis. However, with careful planning and consideration of these limitations, photovoice is a valuable participatory research method that can provide unique insights into the experiences and perspectives of participants.

Our study limitations include the small study sample and the inability to recruit females. The lived experience of others may be more varied. Future research should include a more diverse sample to enhance the generalisability of our findings. Physician selection may have also introduced bias. For example, selecting those perceived as more tech literate or having an interesting story to share. Thus, selection bias may have excluded those with valuable insights or experiences related to our research topic. One way we addressed this is by offering training to participants on how to take and send photos. Finally, the photovoice approach usually concludes with a group discussion so participants can share and reflect on their photos together. Due to COVID-19 restrictions at the time of this study, we could not arrange a physical group meeting. An online discussion was considered, but due to the group’s technological abilities, this was deemed unfeasible.

## Conclusion

We find that photovoice can be an empowering tool, particularly for those who would otherwise struggle to articulate their views in words alone. The impact of COPD on ADLs is complex and diverse. Understanding these lived experiences and the contextual factors that influence them is important for screening and intervention development. More needs to be done to build self-management capabilities outside of COPD rehabilitation, which has been largely unsuccessful in terms of uptake.

### Supplementary Information

**Additional file 1. **

## Data Availability

The dataset analysed during the current study is not publicly available but is available from the corresponding author on reasonable request.
